# Synthesis of a Zinc Hydroxystannate/Sepiolite Hybrid Additive to Avoid Fire Propagation and Reduce Smoke Emission of EPDM Rubber Nanocomposites

**DOI:** 10.3390/ma15186297

**Published:** 2022-09-10

**Authors:** María Luisa Puertas, Teresa Durán, José Florindo Bartolomé, Antonio Esteban-Cubillo

**Affiliations:** 1 Escuela Técnica Superior de Ingenieros Industriales (ETSII), Universidad Politécnica de Madrid (UPM), 28006 Madrid, Spain; 2 Research and Development Department, Tolsa S. A., 28031 Madrid, Spain; 3 Instituto de Ciencia de Materiales de Madrid (ICMM), Consejo Superior de Investigaciones Científicas (CSIC), 28049 Madrid, Spain

**Keywords:** sepiolite, flame retardant, rubber, composite

## Abstract

A zinc hydroxystannate/sepiolite (SEPZHS) hybrid additive was successfully prepared following a facile wet chemical route synthesis where zinc hydroxystannate (ZHS) nanoparticles were grown on the sepiolite’s surface. SEPZHS particles have a fibrillar structure with ZHS nanoparticles homogeneously dispersed and with significantly smaller particle sizes than the synthesized ZHS nanoparticles alone. Sepiolite and SEPZHS were organically modified and introduced in a basic ethylene propylene diene monomer rubber (EPDM) formulation for cable to evaluate the nanocomposite behavior under direct fire sources. The results confirmed the synergistic effect of the hybrid SEPZHS additive in the formation of a most stable and efficient char barrier, thus improving the flame-retardant behavior of EPDM nanocomposite in terms of heat emission, with reductions of more than 40% in the peak of Heat Release Rate (cone calorimeter test), and smoke suppression, with more than 25% reduction in the Total Smoke Production and Smoke Density parameters (smoke chamber test). Moreover, the addition of sepiolite-based additives increased the mechanical properties (hardness) of the nanocomposites, as a result of the matrix reinforcement. This suggests that the SEPZHS hybrid additive may provide a promising option for a new, cost-effective, eco-friendly, yet efficient flame-retardant solution.

## 1. Introduction

Polymeric materials are present, in one way or another, in almost every application nowadays. Textile, packaging, building and construction, cable, transport, electronics, and household devices are some of the applications where polymers have wide use due to their outstanding performance in terms of mechanical properties, chemical, corrosion and aging resistance, lightweight, ease of processing and design possibilities, among others. The widespread use of polymeric materials makes it necessary to ensure safety for users. The resistance to fire is one of the most important aspects related to the safety of these materials owing to their inherent flammability due to their structure and organic composition [1].

The combustion of polymers is a complex multi-step cycle process that involves degradation chemistry with heat transfer and mass diffusion simultaneously [2]. At the combustion step, smoke is released, and its inhalation is known to be the biggest cause of death during a fire rather than burns or heat produced by flames [3]. Additionally, with the use of synthetic polymers replacing natural materials, and using additives, including fire retardants (FRs), to enhance the material properties, the density and toxicity of smoke released in a fire event have increased [4]. The main strategy currently used to achieve the behavior required regarding fire resistance is using FRs and smoke suppressant additives to control fire hazard parameters such as burning heat release, flame spread, smoke production, smoke spread, and smoke toxicity [5,6,7].

In recent years, the safety requirements of polymeric formulations regarding their fire behavior have become stricter. This is the case, for example, of the Fire Protection on Railway Vehicles European Standard (EN 45545) or the Construction Product Regulation (CPR). In addition to this, some flame-retardant solutions, such as some halogenated FRs and synergists, have been already banned or are being watched due to their potential health and environmental risks [8,9,10].

Under this perspective exposed above, synergistic additives to flame retardants are a necessary alternative to achieve the requirements demanded in the regulation, while using solutions that are safe for health and for the environment. Tin-based smoke suppressant additives such as, for example, zinc hydroxystannate, ZnSn(OH)_6_ (ZHS), act as smoke suppressant synergists by the combination of condensed phase mechanism (as a char promoter) and, in halogenated formulations, a vapor phase mechanism based on the volatilization of tin and zinc [11,12,13]. Many examples from the literature show the effectiveness of zinc-tin compounds in different combinations of polymer-FR system [14,15,16,17,18,19]. 

On the other hand, clay-based synergists have proven to be an efficient alternative to achieving the required behavior of polymeric materials [6]. These type of materials act as a reinforcing agent when dispersed into a polymeric matrix and enhance mechanical and barrier properties of carbonaceous char formed during fire, reducing fuel release to the gas phase and, consequently, the heat release rate and smoke rate production [20]. In addition, well-dispersed clays have shown to be a very effective synergist to avoid dripping due to the control of melting flow index during fire conditions [20]. To improve the dispersion of the clay inorganic particles, it is usual to modify the additive surface with organic compounds as quaternary ammonium salts [21]. 

A lot of investigation has been done to study clay FR synergists and, from among all types of clays, montmorillonite (MMT), a layered phyllosilicate that belongs to the dioctahedral smectites group, is probably the most studied and reported [22,23]. Apart from MMT, other types of clays have been also considered and studied as synergists with FR additives for polymers. Halloysite [24,25], a tubular phyllosilicate from the group of kaolinite, and sepiolite [26,27,28,29] a needle-like phyllosilicate due to inverted ribbons at its structure have been also deeply studied and its flame-retardant synergism confirmed. The morphology of the clay is also beneficial for the reinforcement of the composite microstructure [30]. Acicular particles of clay together with lamellar particles, for example, of ATH, present an improved microstructure reducing the porosity and increasing mechanical and barrier properties of the char.

In addition to its flame-retardant synergism and due to the versatility of the chemistry and structure of its surface, sepiolite can be used as a template to grow different particles homogeneously distributed controlling their size and phase of them [31,32]. These particles could be from a wide range of types: metals (Cu, Ag, Au, etc.), oxides (Fe_2_O_3_, ZnO, Ce_2_O, Zr_2_O, etc.), glass particles, etc. So far, no works have been published on the synthesis of smoke suppressants on clay particles and their use as advanced synergistic materials with flame retardants.

In this work, a novel additive based on sepiolite is designed to obtain the benefits of the clay as a flame-retardant synergist while boosting char reinforcement and smoke suppression by synthesizing ZHS nanoparticles homogeneously on its surface. The use of sepiolite as a substrate for particle growth brings the advantage, in comparison with simple particle mixing, of the improvement on the efficiency of ZHS by obtaining smaller particle sizes completely avoiding any agglomeration. This results in a more efficient combination of sepiolite and ZHS FR mechanisms. The effectiveness of the additive has been tested by the preparation of a compound based on ethylene propylene diene monomer rubber (EPDM) and aluminum hydroxide (ATH) as the main flame retardant. To improve the dispersion of the sepiolite-based additives into the polymeric matrix, an organic surface modification was made via silane, which is different from the traditional alternatives that can catalyze the vulcanization process of rubber.

## 2. Materials and Methods

### 2.1. Materials 

Purified and micronized sepiolite grade from Vicálvaro (Madrid), with a sepiolite content higher than 95% and a specific surface of 320 m^2^/g, was prepared by TOLSA (Madrid, Spain). Zinc Sulphate Heptahydrate (99%) and Sodium Stannate Trihydrate (95%) were purchased from Sigma-Aldrich (St. Louis, MO, USA). Sulfuric acid (96%, technical grade) was purchased from Panreac Química (Castellar del Vallès, Spain). Amino-silane (3-Aminopropyltriethoxysilane (>99%), Dynasylan AMEO) was kindly supplied by Evonik (Essen, Germany).

Materials for reference cable formulation were collected as follows: EPDM (Nordel IP 4725P, Dow, Midland, MI, USA) was supplied by Resinex Spain (Vilallonga del Camp, Spain). Aluminum Hydroxide (Martinal OL 107) was supplied by Martinswerk (Huber Engineered Materials, Bergheim, Germany). Peroxide masterbatch formulation (Perkadox 14-40 MB) was supplied by Nouryon (Amsterdam, The Netherlands). Coagent for peroxide crosslinking, Triallyl Cyanurate (TAC) 97% pure, was purchased at Sigma Aldrich. Stearic Acid (40% stearic, 60% palmitic), Paraffinic Oil, and Zinc Oxide (>95% pure) were purchased at Quimipur (Campo Real, Spain).

### 2.2. Synthesis of ZHS Particles

A single synthesis of zinc hydroxystannate particles was performed using zinc sulphate and sodium stannate as precursors. The reasons why this synthesis was carried out were, on the one hand, to establish the most suitable reaction conditions for the preparation of the SEPZHS hybrid additive and, on the other hand, to obtain a reference to compare and evaluate the particle size and homogeneity of ZHS nanoparticles synthesized if using the clay as a substrate with abundant nucleation centers available. The synthesis occurs through the following reaction:

ZnSO_4_ + Na_2_SnO_3_ + 3H_2_O → ↓ZnSn(OH)_6_ + Na_2_SO_4_

ZHS precursors (ZnSO_4_ and Na_2_SnO_3_) were dissolved using a magnetic stirrer in deionized water (5 g in 100 mL of solution). Solution of Na_2_SnO_3_ was added slowly drop by drop into the ZnSO_4_ solution, under magnetic stirring. A white precipitate appeared while adding the stannate solution and increased its presence with pH evolution. Once sodium stannate was completely added, stirring remained for 24 h and then, the solution was centrifuged at 5.000 rpm. Solid obtained (approx. 5 g) was dried at 80 °C for 4 h.

### 2.3. Synthesis of SEPZHS Hybrid Particles

**Figure 1:**
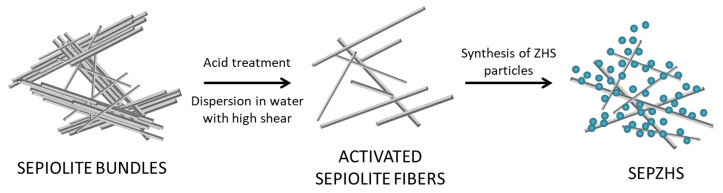
Scheme for the sepiolite additive with ZHS nanoparticles preparation.

To obtain the joint effect between sepiolite and nanoparticles of ZHS, SEPZHS additive has been prepared following the process schemed in Figure 1.

To prepare the additive SEPZHS, sepiolite was dispersed in an H_2_SO_4_ solution 0.1 M, using a Cowles mixer and applying high shear (18 m/s) in order to obtain a homogeneous dispersion of individualized sepiolite fibers. Dispersion in acid media leaches magnesium cations from sepiolite’s structure borders forming silanol groups that act as active spots for particle growth [33]. A sepiolite/ZHS weight ratio of 75/25 was selected for the synthesis based on previous works [34,35]. For this selection it was also considered, on the one hand, to cover the sepiolite surface fully and homogeneously with the particles and, on the other, to incorporate enough ZHS to significantly have an effect in the application, enhancing the flame-retardant synergistic behavior of sepiolite.

Synthesis of ZHS particles was done following the route explained previously in Section 2.2. Obtained dispersion was filtered and dried at 80 °C for 4 h.

### 2.4. Preparation of EPDM Fire Retardant Compounds

To test the nanocomposites’ efficacy against fire, EPDM compounds were prepared based on a cable formulation shown in Table 1. SEPZHS and sepiolite were both organically modified with a silane to enhance compatibility with EPDM. Organic modification with amino-silane was carried out, at a concentration of 4.5 mmol per gram of raw sepiolite, following a procedure described by García et al. [36]. Both additives, renamed OSepZHS and OSep to differentiate from the organically unmodified materials, were added on top of the formulation at a dosage of 3 wt% (8.5 parts per hundred rubber (phr)). This dosage was selected considering the typical dosage of clay-based synergists used for cable formulations to improve their fire-retardant properties (≤5 wt%) [37,38], and based on previous own experience and studies, as the work based on a polymer-metal hydrate system carried out by Huang et al. [39] where best-balanced results for fire retardant performance were obtained with 3 wt% of sepiolite.

For the compound preparation, components were processed in a Banbury mixer at 160 °C with a rotor speed of 60 rpm. After all components were incorporated, mixture was homogenized in an open two-roll mill where a vulcanization system was added. Compounds were vulcanized in a hot plate press at 180 °C during optimum vulcanization time (t95, time required to achieve 95% of the maximum torque), previously determined by a moving die rheometer (Monsanto MDR 2000E). It has been confirmed that the sepiolite-based additives do not affect significatively the vulcanization parameters (Table 2).

### 2.5. Characterization

The crystalline structure was analyzed by a Bruker AXS D8 Advanced (Billerica, MA, USA) X-ray diffraction spectrometer (XRD) using Cu Kα radiation (λ = 0.15042 nm) generated at 40 kV and 35 mA. The microstructure of the materials was studied by field emission scanning electron microscopy (FSEM Nova NanoSEM 230, FEI, Hillsboro, OR, USA) with energy-dispersive X-ray spectroscopy (EDX, EDAX Génesis XM2i) for quantitative chemical microanalysis of samples. Transmission electron microscopy (TEM) was performed using a JEOL FXII (Tokyo, Japan). TEM images were obtained at 120 kV.

EPDM compounds were characterized against fire using a cone calorimeter (FTT, East Grinstead, UK), following the procedures in ISO5660-1 at a heat flux of 50 kW/m^2^. Square specimens (100 × 100 × 4 mm^3^) were wrapped with aluminum foil and placed over refractory fiber material. A retention frame with 4 retention metallic cables to avoid sample deformation was used. The distance between the specimen surface and the radiation source was 25 mm. Vertical burning tests were conducted according to UL-94 standard on a vertical burning test instrument with sheet dimensions of 130 × 13 × 3.2 mm^3^ and performing 5 repetitions. A smoke chamber test according to ISO 5659-2 was performed at a heat flux of 50 kW/m^2^ with a distance between the specimen surface and the radiation cone of 25 mm. Smoke density is determined by means of the transmission measured between a photo-emissor and a photodetector placed inside the chamber. Squared specimens (75 × 75 × 4 mm^3^) were wrapped in aluminum foil, tested using a retention frame, and placed directly over a calcium silicate plate that is, at the same time, over refractory fiber material. Tests had a duration of 1200 s and were run in triplicate. Test specimens for cone calorimeter and smoke chamber tests were conditioned until constant weight (variation less than 0.1% in 24 h) in a controlled atmosphere (23 ± 2 °C and 50% ± 5% of relative humidity). International Rubber Hardness Degree (IRHD, resistance of a rubber specimen against the intrusion of a spheric indentor) was measured in a Wallace Rubber Hardness Tester (Dorking, UK). 

## 3. Results and Discussion

### 3.1. Synthesis and Characterization of ZHS and SEPZHS

**Figure 2:**
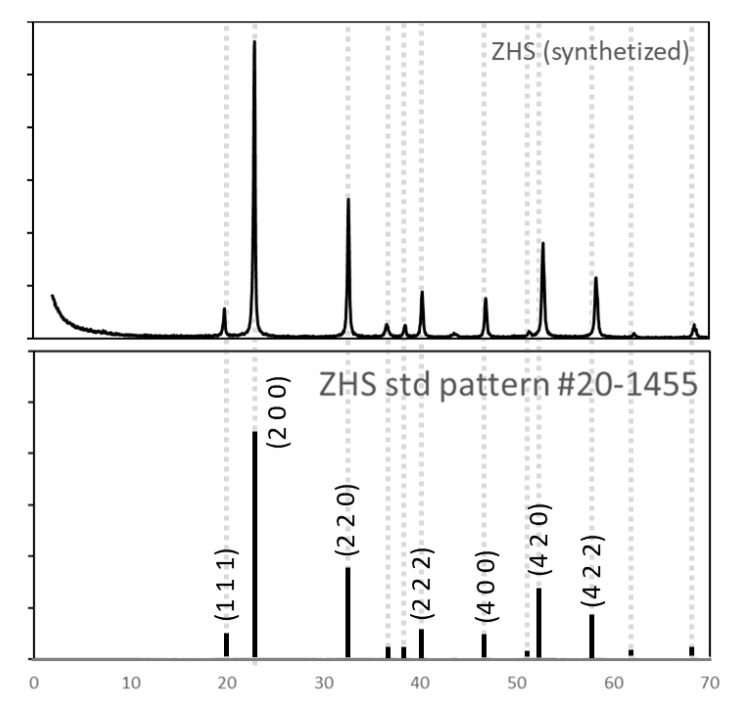
X-ray diffractograms of ZHS synthesized, compared with standard pattern (ICDD/JCPDS, PDF no. 20-1455).

Obtained particles were characterized by XRD (Figure 2) and compared with International Centre for Diffraction Data (ICCD/JCPDS, PDF no. 20-1455) [40]. Comparison between diffractograms concludes that particles obtained exhibit high crystallinity and correspond to ZHS structure while no other crystalline structures are present in such amount to be detected by X-ray diffraction characterization performed.

**Figure 3:**
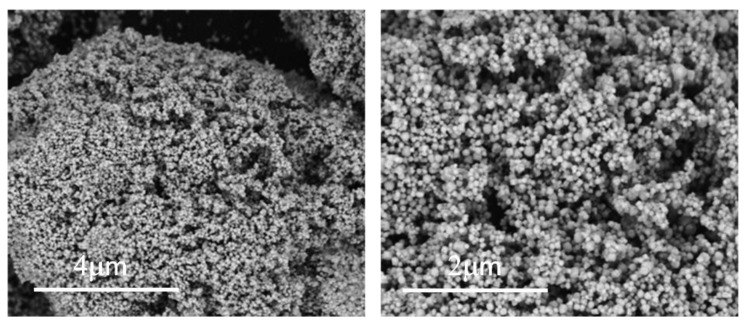
FE-SEM micrographs of synthesized ZHS nanoparticles at 30 k and 60 k magnification.

ZHS particles obtained have been characterized in powder form after the synthesis by FE-SEM (Figure 3). Soft/fluffy agglomerates of cubic ZHS nanoparticles, with sizes between 50 and 250 nm, can be observed in the micrographs.

**Figure 4:**
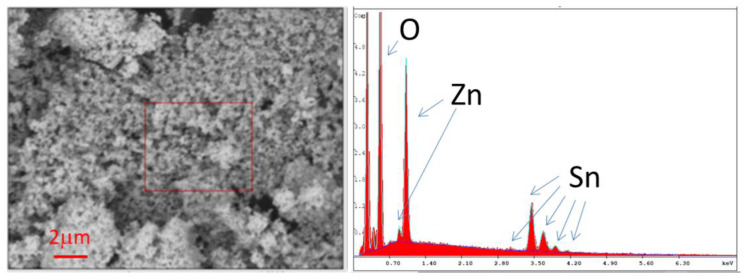
EDS analysis and spectrum for synthesized ZHS.

Figure 4 shows an EDS spectrum from the particle agglomerates. The EDS spectrum suggests that the atomic ratio of zinc to tin to oxygen is approximately 1:1:6 (within experimental error), implying the stoichiometry of ZHS (ZnSn(OH)_6_).

**Figure 5:**
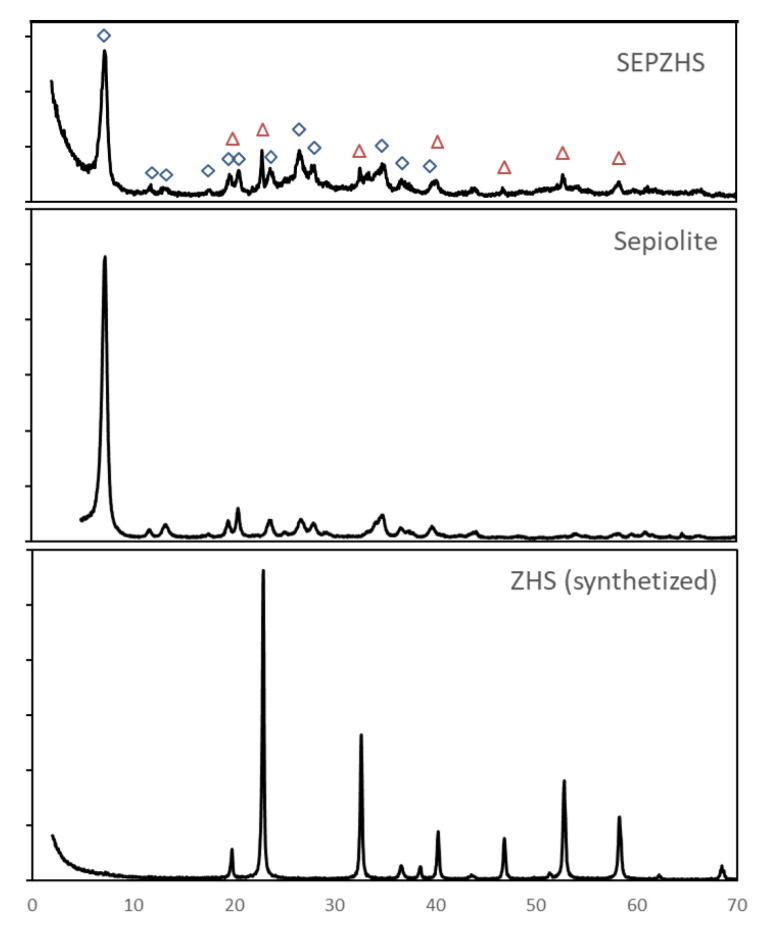
XRD for SEPZHS additive, sepiolite, and ZHS synthesized. Corresponding peaks of sepiolite (◊) and ZHS (∆) are marked in the SEPZHS diffractogram.

**Figure 6:**
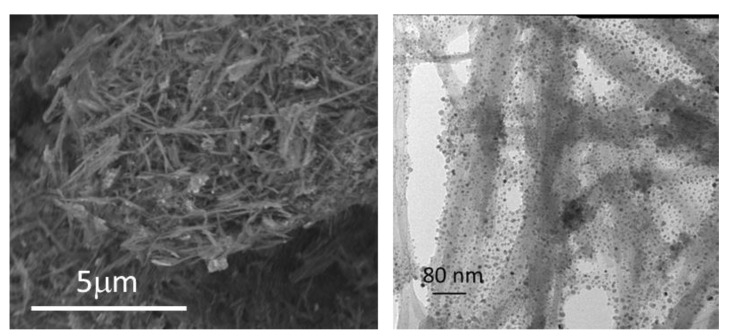
FE-SEM (**left**) and TEM (**right**) micrographs of additive SEPZHS.

SEPZHS was characterized by XRD, FE-SEM, and TEM. Diffractograms (Figure 5) show that particles of ZHS are present but peaks in SEPZHS have less intensity than the peaks in ZHS (synthesized) diffractogram. The activated surface of the sepiolite, with more spots for particle nucleation and growth, could have led to much smaller ZHS particles in additive SEPZHS and this could be the cause of the differences between peak intensities. Homogeneous distribution and size of the particles can be observed in FE-SEM and TEM micrographs (Figure 6). It can also be observed much smaller particles in micrographs of additive SEPZHS than the particles observed for ZHS in Figure 3, confirming the explanation of the differences observed in the XRD comparison between peaks intensities.

### 3.2. Characterization of EPDM Compounds

#### 3.2.1. Char Stability Characterization

**Figure 7:**
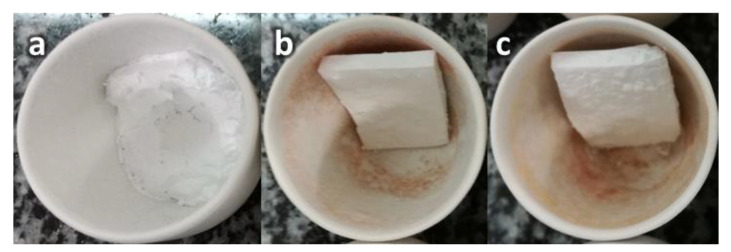
EDPM chars obtained after thermal treatment: (**a**) EPDM Reference, (**b**) EPDM OSep, (**c**) EPDM OSepZHS.

To check the influence of the nanocomposite sepiolite-based additives in char formation for EPDM formulations, pieces of the compound were cut and placed into porcelain crucibles and then treated in an oven at 1000 °C for 1 h. In Figure 7, the chars obtained are compared. It can be observed that reference char does not maintain its dimensional stability and it is mechanically weaker than the ones with sepiolite-based additives.

#### 3.2.2. Vertical Burning Test Based on UL-94

**Figure 8:**
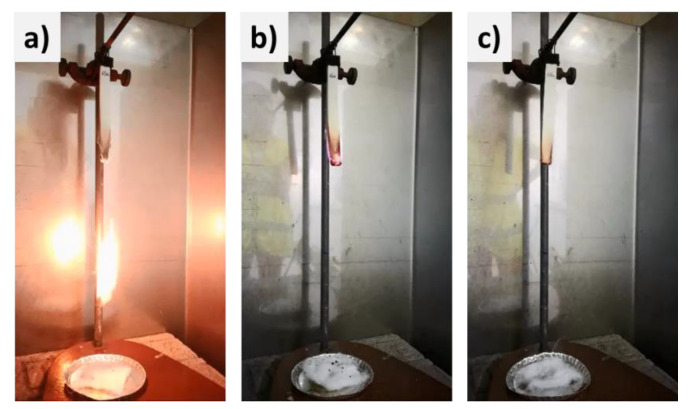
EDPM probes during vertical burning test. (**a**) EPDM Reference, polymer drips and burns the cotton 51 s after third flame application; (**b**) EPDM OSep, polymer burns for more than 60 s without dripping; (**c**) EPDM OSepZHS, polymer fades out at 4 s after third flame application without dripping.

All the EPDM formulations presented an excellent resistance to fire due to the high content of inorganics, passing UL-94 without flaming dripping and very short burning times (less than 10 s) after two applications of a controlled flame to the test bar for 10 s (V-0 rating) To find differences between formulations, an additional third flame exposure of 10 s was performed. Illustrative photographs taken during vertical burning tests are shown in Figure 8. Results obtained showed that, after the third flame application, the reference formulation was not able to extinguish the flame in a short time and, as a result, the polymer dripped and burnt the cotton (Figure 8a). Burning times after third flame applications (t3) in all five repetitions for reference formulation were between 39 and 51 s. Formulation with OSep avoided the dripping due to the enhanced viscosity and char resistance achieved by the presence of sepiolite particles. On the other hand, the burning time was longer than the reference after the third flame application and t3 for all repetitions exceeded 60 s (Figure 8b). This can be attributed to the reduction of the water release capacity of ATH due to the higher viscosity of the melt polymer. Formulation with OSepZHS showed enhanced behavior avoiding dripping and flame propagation. Burning times from the end of the flame application, t3, were much shorter taking only a few seconds, between three and seven for all repetitions, to stop the flame (Figure 8c). The improved char reinforcement by the presence of ZHS nanoparticles coupled with the additional effect on the condensed phase by water release, gives the material the capacity for self-extinction.

#### 3.2.3. Cone Calorimeter and Smoke Chamber Tests

**Figure 9:**
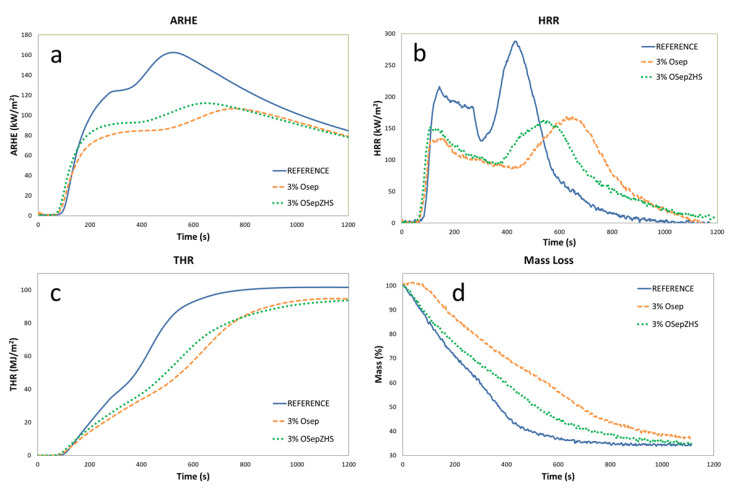
Cone calorimeter results: (**a**) ARHE, (**b**) HRR, (**c**) THR, and (**d**) Mass Loss curves.

**Figure 10:**
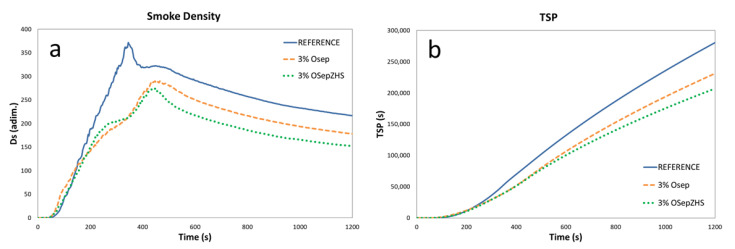
Smoke chamber test results: (**a**) Smoke Density (Ds) and (**b**) Total Smoke production (TSP) curves.

Cone calorimeter and smoke chamber test results are shown in Table 3 and Table 4 and Figure 9 and Figure 10. All formulations with sepiolite-based additives improved the fire behavior of the reference reducing the Maximum Average Rate of Heat Emission (MARHE) in more than 30% and, in more than 40%, the peak of heat release rate (pHRR). No significant improvements were observed in the Total Heat Release (THR) parameter at the end of the test (1200 s) but, analyzing the graph in Figure 9c, an important heat release delay in time is observed. For example, at 600 s from the test start, the total heat released by sepiolite-containing formulations is 30% less than the heat released by the reference formulation. This means that the barrier char reinforced by sepiolite additives, which was previously evidenced by char stability test results in Section 3.2.1, slow down the intensity and spread rate of fire; this is also observed analyzing mass loss curves in Figure 9d, where reference mass loss is faster in comparison with formulations with sepiolite-based additives.

Although no significant differences were observed between OSep and OSepZHS formulations in cone calorimeter results, better performance of OSepZHS is shown in smoke chamber test results. With the addition of OSep in the EPDM formulations, a decrease of 21% in total smoke production after 1200 s (TSP 1200 s), a decrease of 17% in the maximum specific optical density obtained within the 20 min test period (Ds max 20′) and a decrease of 10% in VOF4 parameter (a measurement of the rate of smoke production during the first four minutes of the test) are achieved. When OSepZHS additive is incorporated, further reductions of total smoke production, specific optical density, and VOF4 are achieved (decreases of 26%, 26%, and 13%, respectively, in comparison with the reference). These positive outcomes were due to the formation of a compact char protective layer where SEP and ZHS work synergistically taking advantage of ZHS particles as a smoke suppressor [16]. 

#### 3.2.4. Mechanical and Microstructural Properties of the Compounds

The hardness (IRHD) of the compounds was tested obtaining 81 ± 1, 84 ± 1, and 83 ± 1 as values for REFERENCE, 3% OSep, and 3% OSepZHS, respectively. The addition of sepiolite-based additives slightly increases the material hardness due to the mechanical reinforcement because of the presence of sepiolite particles and also to the sepiolite’s capacity to absorb the oil from the formulation. In OSepZHS additive, the available surface and sepiolite quantity incorporated is less than in OSep, so this increase in hardness is observed to a lesser extent.

**Figure 11:**
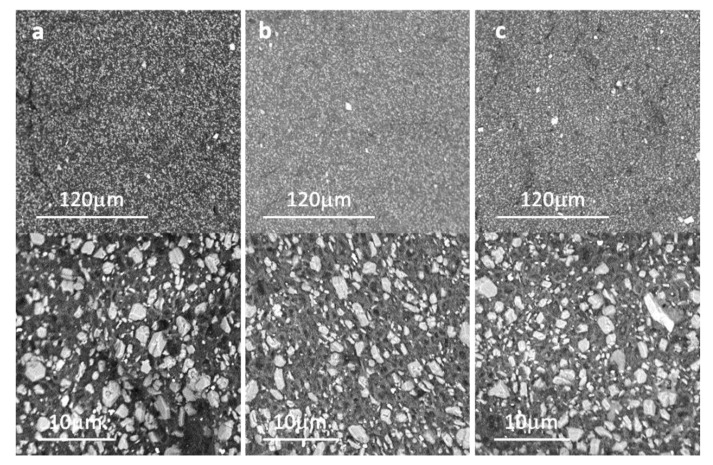
FE-SEM micrographs of EPDM compounds: (**a**) EPDM Reference, (**b**) EPDM OSep and (**c**) EPDM OSepZHS.

Reinforcement of materials is a confirmation that the different phases have been correctly dispersed into the polymeric matrix, which is not trivial [41]. To check the dispersion of additives, the fracture surface of the compounds was observed by FE-SEM (Figure 11). Sepiolite-based additives individual fibrils have lengths between 1 and 2 μm with the other two dimensions of the particle on the nanoscale, which makes observation of additives dispersion within the polymer matrix very difficult and even more in these kinds of highly filled formulations containing other particles of different shapes and sizes. On the contrary, sepiolite agglomerates are relatively easy to distinguish from other particles in the formulation because of their different shape and texture. To check sepiolite dispersion, we focused our observations on finding these kinds of agglomerates and, as can be seen in the representative micrographs in Figure 11, no big agglomerates are present so it can be assumed that a good dispersion has been achieved. ATH particles (white phase) uniformly distributed in the polymeric matrix can be observed.

## 4. Conclusions

Zinc hydroxystannate/sepiolite hybrid additive with homogeneous nanoparticle distribution has been successfully prepared. The incorporation of 3 wt% of this additive into the EPDM polymer matrix led to an important heat release delay in time and a significant reduction of the pHRR values (40%), compared to those of reference EPDM. This means that barrier char generated with sepiolite-based additives slow down the intensity and spread rate of fire. Additionally, the amount of smoke released during the combustion in terms of density and production was significantly reduced. The notable reduction of the fire hazard was mainly attributed to the improved char formation due to the synergy between sepiolite and ZHS synthesized nanoparticles. In addition, sepiolite-based additives slightly increased the hardness of the EPDM materials without significant interference in the vulcanization process.

## Figures and Tables

**Table 1:** Formulation of EPDM system for cable. The composition is shown in phr (parts per hundred rubber).

Components	REFERENCE	3% OSep	3% OSepZHS
EPDM-Nordel 4725	100	100	100
Stearic Acid	1	1	1
ATH	160	160	160
ZnO	5	5	5
Paraffinic Oil	10	10	10
Perkadox 14-40 MB	5	5	5
TAC	1.5	1.5	1.5
OSep	-	8.5	-
OSepZHS	-	-	8.5

**Table 2:** Vulcanization parameters for EPDM compounds.

	Min Torque (dNm)	Max Torque (dNm)	t5 (min)	t50 (min)	t90 (min)
REFERENCE	1.04	44.77	0.41	1.24	3.57
3% OSep	1.59	45.49	0.35	1.16	3.42
3% OSepZHS	1.25	44.92	0.40	1.23	3.48

**Table 3:** Cone calorimeter and smoke chamber results. MARHE (kW/m^2^): Maximum Average Rate of Heat Emission. THR 1200 s (MJ/m^2^): Total Heat Release. pHRR(kW/m^2^): Peak of heat release rate.

	MARHE (kW/m^2^)	THR 1200 s (MJ/m^2^)	pHRR (kW/m^2^)
REFERENCE	162 ± 8	101 ± 5	288 ± 4
3% OSep	107 ± 5	95 ± 3	168 ± 7
3% OSepZHS	112 ± 5	94 ± 4	163 ± 9

**Table 4:** Smoke chamber test results. TSP 1200 s: total smoke production after 1200 s, Ds max (20′): maximum specific optical density obtained within the 20 min test period. VOF4: parameter of the rate of smoke production during the first four minutes of the test.

	TSP (1200 s)	Ds Max (20’)	VOF4
REFERENCE	4676 ± 23	371 ± 11	339 ± 9
3% OSep	3849 ± 36	290 ± 6	302 ± 13
3% OSepZHS	3452 ± 24	274 ± 9	293 ± 10

## Data Availability

The raw/processed data required to reproduce these findings cannot be shared at this time as the data also forms part of an ongoing study.
